# Gu-4 Suppresses Affinity and Avidity Modulation of CD11b and Improves the Outcome of Mice with Endotoxemia and Sepsis

**DOI:** 10.1371/journal.pone.0030110

**Published:** 2012-02-02

**Authors:** TingTing Yan, Qing Li, HuiTing Zhou, YueTao Zhao, ShuQin Yu, GuangLin Xu, ZhiMin Yin, ZhongJun Li, ZhiHui Zhao

**Affiliations:** 1 Jiangsu Province Key Laboratory for Molecular and Medical Biotechnology, College of Life Science, Nanjing Normal University, Jiangsu Province, China; 2 State Key Laboratory of Natural and Biomimetic Drugs, School of Pharmaceutical Sciences, Peking University, Beijing, China; Brigham and Women's Hospital, United States of America

## Abstract

**Background:**

Systemic leukocyte activation and disseminated leukocyte adhesion will impair the microcirculation and cause severe decrements in tissue perfusion and organ function in the process of severe sepsis. Gu-4, a lactosyl derivative, could selectively target CD11b to exert therapeutic effect in a rat model of severe burn shock. Here, we addressed whether Gu-4 could render protective effects on septic animals.

**Methodology/Principal Findings:**

On a murine model of endotoxemia induced by lipopolysaccharide (LPS), we found that the median effective dose (ED50) of Gu-4 was 0.929 mg/kg. *In vivo* treatment of Gu-4 after LPS challenge prominently attenuated LPS-induced lung injury and decreased lactic acid level in lung tissue. Using the ED50 of Gu-4, we also demonstrated that Gu-4 treatment significantly improved the survival rate of animals underwent sepsis induced by cecal ligation and puncture. By adhesion and transwell migration assays, we found that Gu-4 treatment inhibited the adhesion and transendothelial migration of LPS-stimulated THP-1 cells. By flow cytometry and microscopy, we demonstrated that Gu-4 treatment inhibited the exposure of active I-domain and the cluster formation of CD11b on the LPS-stimulated polymorphonuclear leukocytes. Western blot analyses further revealed that Gu-4 treatment markedly inhibited the activation of spleen tyrosine kinase in LPS-stimulated THP-1 cells.

**Conclusions/Significance:**

Gu-4 improves the survival of mice underwent endotoxemia and sepsis, our *in vitro* investigations indicate that the possible underlying mechanism might involve the modulations of the affinity and avidity of CD11b on the leukocyte. Our findings shed light on the potential use of Gu-4, an interacting compound to CD11b, in the treatment of sepsis and septic shock.

## Introduction

Sepsis, severe sepsis and septic shock, the leading causes of death in surgical intensive care unit patients, are characterized by pathological changes within the microcirculation [1]. Impairment of the microcirculation causes severe decrements in tissue perfusion and organ function and plays a vital role in the progression to severe sepsis [2], [3]. Observations from various experimental models of sepsis, such as endotoxemia and cecal ligation and puncture (CLP), have suggested that the representative event that takes place within microcirculation is the increase of rolling and adhering leukocytes in small blood vessels [4], [5], [6]. Hence, systemic leukocyte activation and disseminated leukocyte adhesion are regarded to be essential for septic organ dysfunction.

Many phagocytic components (neutrophils/macrophage/monocytes) of the immune system as well as non-immune cells (epithelia, endothelia, etc.) express TLR4 and its associated lipopolysaccharide (LPS) receptor complex [7]. When challenged by LPS, the component of the outer membrane of Gram-negative bacteria, this phagocytic system will respond with an exaggerated inflammatory mediator release. In turn, inflammatory mediators together with LPS induce the upregulation of adhesion molecules and promote the adhesion of leukocytes to endothelium [8], [9]. The initial capture and rolling of leukocytes on the activated endothelium are mediated by selectins. Selectin-interactions and local chemokines activate leukocyte integrins, such as lymphocyte function-associated antigen-1 (LFA-1, CD11a/CD18) and macrophage antigen-1 (MAC-1, CD11b/CD18), which favour interactions with endothelial counter-receptors, such as intercellular adhesion molecule-1 (ICAM-1), resulting in a firm adhesion [10], [11].

Because of their direct linkage with the consequences of sepsis, adhesion molecules that mediate leukocyte adhesion become ideal drugable targets for treating this disease [12], [13]. Numerous antagonists directed at adhesion molecules have been prepared and some of them showed promising therapeutic effects in clinical trials for inflammatory diseases [14]. Unfortunately, so far there is no effective antagonist against adhesion molecule has been used for the treatment of sepsis. The currently recommended therapies for the treatment of sepsis are multifaceted, including timely diagnosis, early antimicrobial therapy, ventilation, goal-directed hemodynamic support, targeted immunological therapy and effective supportive therapies. Despite timely intervention, these treatment strategies are unable to abrogate sepsis pathophysiology, and sepsis associated mortality rates remain unacceptably high [15], [16].

Our earlier studies have demonstrated that a lactosyl derivative named Gu-4 (N-[2-(1, 3-dilactosyl)-propanyl]-2-amino-pentandiamide) could selectively target CD11b to exert therapeutic effect in a rat model of severe burn shock [17]. However, whether this tetravalent lactoside could benefit other inflammatory diseases is not clear and, importantly, the precise role of Gu-4 in the interfering with the function of CD11b remains largely unknown. In the present study, we extensively investigated the therapeutic effects of Gu-4 in a murine lethal endotoxemia model induced by LPS and in a sepsis model induced by CLP. We found that Gu-4 obviously protected animals from mortality caused by septic shock, and our findings from *in vitro* experiments suggested that the decrease of the affinity and avidity of CD11b may be one of the possible underlying mechanisms.

## Results

### Gu-4 increases survival rate but does not affect expression profiles of plasma TNF-α and IL-10 in LPS-induced shock model

**Figure 1:**
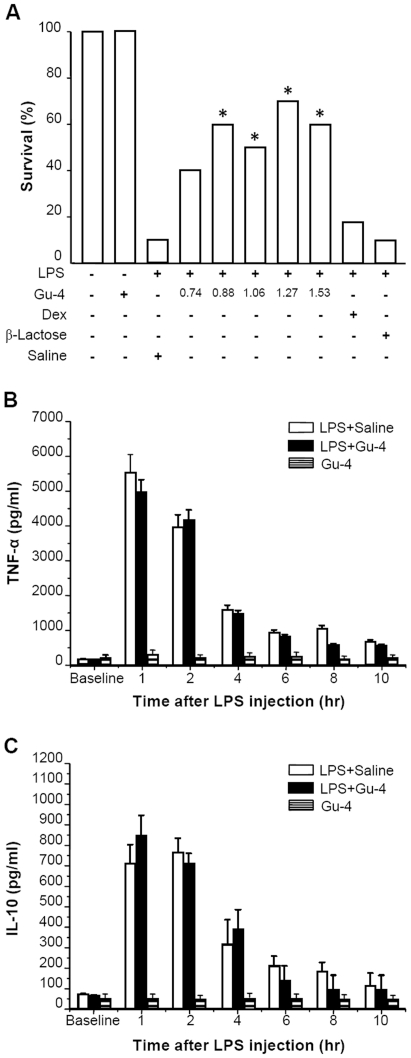
Effects of Gu-4 on survival and expression profiles of TNF-α and IL-10 in septic mice. A: Survival of septic mice induced by LPS stimulation (37.5 mg/kg *i.p.*) (n = 10 in each group) was monitored for 72 hrs. Gu-4 administration significantly reduced LPS-induced mortality at the doses of 0.88, 1.06, 1.27 and 1.53 mg/kg. *: p<0.05 indicates a significant decrease in mortality by Gu-4 treatment versus saline treatment as determined by log-rank test. β-lactose or dexamethasone (Dex) did not produce significant improvement in survival. B and C: Serum levels of TNF-α and IL-10 were measured in groups of mice killed at 0 (baseline), 1, 2, 4, 6, 8 and 10 hr after LPS stimulation, respectively. Both TNF-α (B) and IL-10 (C) productions increased to the maximum level at about 1 hr after LPS injection and then declined. Administration of Gu-4 at the ED50 dose of 0.929 mg/kg did not affect TNF-α or IL-10 expression profile in serum. Data shown represent mean ± SD of n = 10 animals at each time point. Gu-4-alone represents that the mice treated with Gu-4 alone at the dose of 10 mg/kg. No adverse effects were observed in this group (i.e., 100% survival), and Gu-4 treatment at this dose did not affect baseline level of TNF-α or IL-10.

To investigate the role of Gu-4 in protecting mice from endotoxemia and to determine the median effective dose (ED50) of Gu-4 for the survival of LPS-induced endotoxemic mice, we performed a dose-response study with incremental doses of Gu-4 (1.2 fold increments). As shown in Figure 1A, the starting dose of Gu-4 tested (0.74 mg/kg) had no effect, while the following doses markedly improved the survival (p<0.05 *vs.* saline control) and at dose of 1.27 mg/kg, Gu-4 exhibited the highest efficacy. The ED50 of Gu-4 for this murine model is 0.929 mg/kg as estimated using Probit analysis. In addition, Gu-4 treatment alone did not show any hazardous effect on mice. By contrast, neither β-lactose nor dexamethasone showed significant protective effect on endotoxemic mice (p>0.05 *vs.* saline control).

Tumour necrosis factor α (TNF-α) is a pro-inflammatory cytotoxin that has been implicated in septic shock, while interleukin-10 (IL-10) is an anti-inflammatory cytokine capable of inhibiting synthesis of TNF-α [18], [19], [20]. Therefore, we determined the effect of Gu-4 on the expression profiles of circulating TNF-α and IL-10 in the LPS-induced endotoxemic mice. As shown in Figure 1B and C, both the plasma TNF-α and IL-10 increased to the maximum level at about 1 hr after LPS challenge, and declined gradually thereafter. In endotoxemic mice treated with Gu-4, there was no obvious change in the expression profiles of TNF-α and IL-10 at the dose of 0.929 mg/kg, indicating that Gu-4 achieves its survival benefit through some other ways but not through affecting the expression of TNF-α or IL-10 in serum.

### Gu-4 attenuates the organ injury and the increase of LA level in lung of LPS-induced shock model

**Figure 2:**
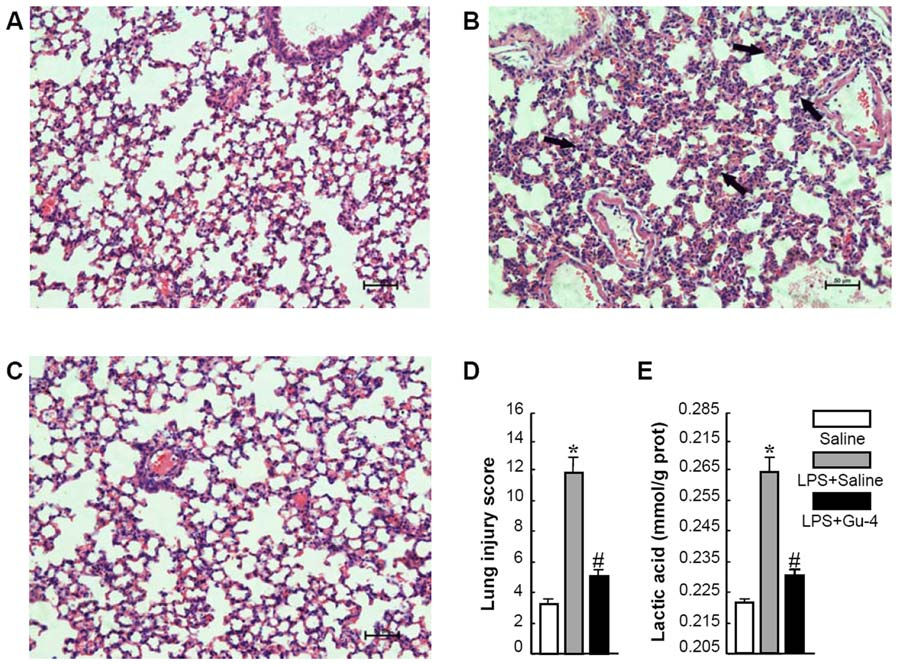
Effects of Gu-4 on lung injury and LA level of lung tissue in septic mice. A: Lung tissues from saline-treated mice exhibited normal architectures. B: Lung tissues from LPS-challenged mice showed severe edema and leukocyte infiltration as indicated with black arrows. C: Lung tissues from Gu-4-treated LPS-challenged mice showed normal architectures and significant decrease of inflammatory cell infiltration. D: Semi-quantitative scoring of lung injury was determined as described in Materials and Methods. The lung injury was seriously induced by LPS but was attenuated by Gu-4. E: LA level in lung tissue from septic mice is significantly higher than those from normal mice and Gu-4-treated LPS- challenged mice. Magnification: ×200. *: p<0.05 *vs*. Normal; #: p<0.05 *vs*. LPS + Saline.

Among the vital organs in the body, the lung is particularly susceptible to acute injury in LPS- induced endotoxemia [21]. To determine whether Gu-4 could protect mice underwent septic shock from acute acute injury, we performed histological examinations and analyzed the effect of Gu-4 treatment on leukocyte infiltration in lung tissues. In comparison with the architectures of lung from saline treated normal mice (Figure 2A), lung tissues from LPS-treated mice exhibited severe edema and infiltration of the inflammatory cells and granulocytes (Figure 2B). By contrast, granulocytes infiltration and edema were obviously attenuated in lungs after *in vivo* administration of Gu-4 (0.929 mg/kg, *i.v.*) (Figure 2C). We then evaluated the lung injury on histological sections using a semi-quantitative scale (detailed described in Materials and Methods). As shown in Figure 2D, the total injury score in lungs after LPS challenge increased dramatically compared to saline treated ones, while the score decreased markedly almost to the normal level after *in vivo* administration of Gu-4.

Since severe histological damage and leukocytes infiltration resulted from LPS challenge could lead to metabolic deterioration [22], [23], we thus examined the lactic acid (LA) level in lungs of mice with different treatments. As shown in Figure 2E, LPS stimulation for 10 hrs induced a statistically significant elevation of LA level compared to control treatment (p<0.05, LPS *vs*. saline challenge), while the increase of LA level was significantly attenuated after the administration of Gu-4 to the LPS challenged mice (p<0.05, Gu-4 *vs.* LPS).

### Gu-4 blocks the adhesion and transendothelial migration of THP-1 cells

**Figure 3:**
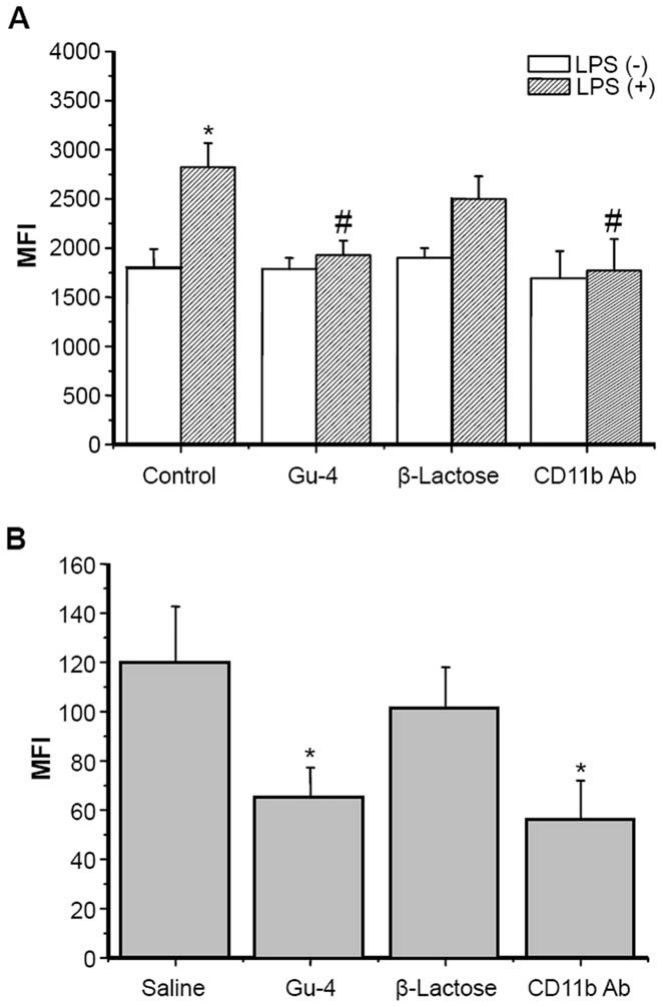
Gu-4 inhibited the adhesion and subsequent transendothelial migration of THP-1 cells. HUVECs and calcein AM-labeled THP-1 cells were pretreated as described in Material and methods and subjected to adhesion and migration assays. A: In adhesion assay, THP-1 cells with LPS stimulation showed enhanced adhesion to HUVECs, whereas THP-1 cells treated with Gu-4 or CD11b antibody, but not β-lactose, after LPS stimulation showed no significant increase of adhesion compared with control cells. The experiments were performed in triplicate. *: p<0.05 *vs.* LPS (−) control. #: p<0.05 *vs.* LPS (+) control. B: In migration assay, LPS-stimulated THP-1 cells were subjected to treatments of Gu-4, β-lactose, CD11b antibody, or saline, and then seeded into inserts with HUVECs monolayer on the outer side of the membrane. The relative amount of migrated cells in the lower chamber were determined by measurement of fluorescence intensity. Both Gu-4 and CD11b antibody significantly reduced the migration of THP-1 cells across HUVECs monolayer. The experiments were performed in triplicate. *: p<0.05 *vs.* saline.

Since histological examinations showed that Gu-4 attenuated the leukocytes infiltration, we thus employed *in vitro* adhesion and transwell assays to deeply investigate the activity of Gu-4. In adhesion assay, the adhesion of LPS-untreated THP-1 cells to HUVECs appeared no observable difference among different treatments, namely, treatment of saline, Gu-4, β-lactose, or treatment of CD11b antibody (Figure 3A). By contrast, upon LPS stimulation, THP-1 cells exhibited an obvious increase in adhesion. Markedly, the adhesive activities of cells treated with Gu-4 and CD11b antibody, but not β-lactose, dramatically decreased (Figure 3A).

The results of transwell experiments were consistent with those observed in adhesion assay. In response to a chemotactic gradient of IL-8, a nearly 50% reduction of migration activity was achieved in THP-1 cells treated with Gu-4 or CD11b antibody as compared to those cells treated with saline (Figure 3B). No obvious difference in cell migration activities was observed between the β-lactose treatment and saline treatment.

### Effects of Gu-4 on the expression and distribution of CD11b

**Figure 4:**
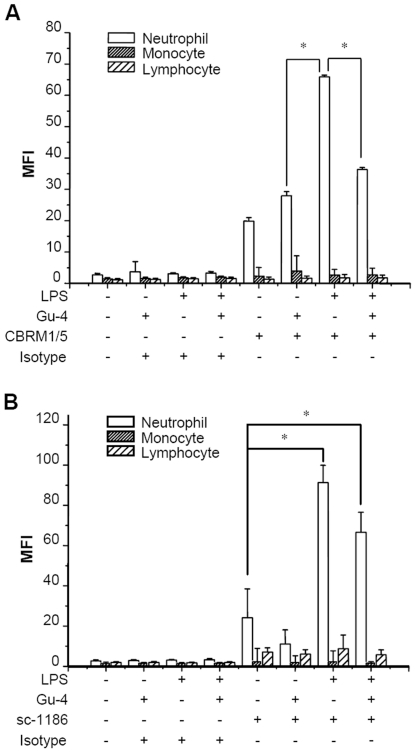
Effects of Gu-4 on the expression of CD11b and the exposure of CD11b active I-domain. In the presence or absence of Gu-4 (40 nmol/ml), whole blood samples from healthy donors were firstly stimulated with or without LPS (100 ng/ml) for 30 mins, then the samples were co-incubated with saturating amounts of PE conjugated CBRM1/5 or PE labeled anti-CD11b antibody sc-1186 for 10 mins, respectively. After removal of red blood cells, different populations of leukocytes were identified by flow cytometry. A: Expression of CD11b active I-domain. Upon LPS stimulation, CD11b active I-domain on neutrophils was greatly increased, but could be markedly supressed by Gu-4 treatment. B: Expression of CD11b. CD11b expression was significantly elevated in LPS-stimulated neutrophils. Gu-4 showed weak inhibitory effect on such elevation. Data represented the results of triplicate experiments. *: p<0.05.

**Figure 5:**
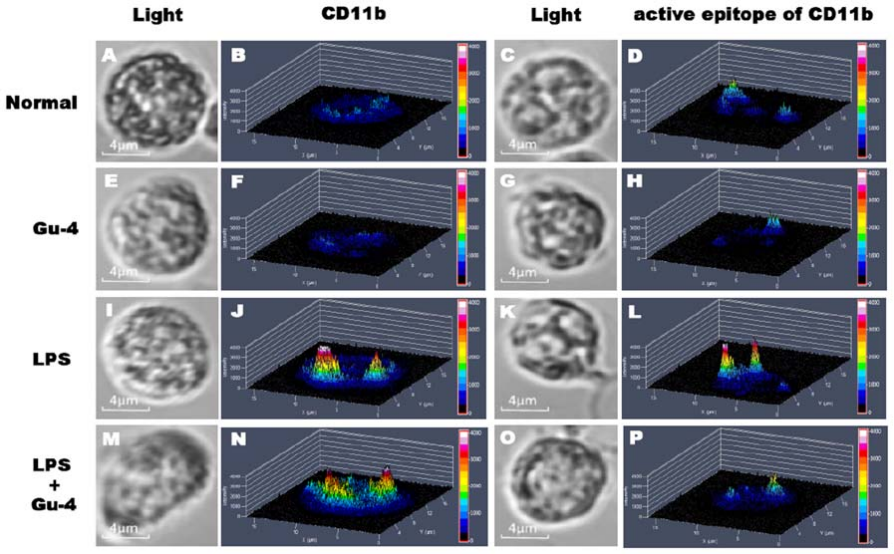
Effects of Gu-4 on the distribution of CD11b and CD11b active I-domain on leukocytes. Leukocytes obtained and treated as described in flow cytometry study were further incubated with Qdot 655 secondary antibody and observed by using a laser scanning confocal microscope. Cells with diameters about 10 µm were imaged and displayed in a pseudo 3D mode (2.5D filled view). Images in the panels of “light” show representative cells acquired under bright field, and those in the other two panels illustrate the mesh plots of fluorescence intensity *vs*. spatial distribution of CD11b and its active I-domain on the corresponding cell in the juxtaposed image. Gu-4 greatly diminished the exposure of CD11b active I-domain in LPS-stimulated leukocyte (P *vs.* L). The increased expression of CD11b in LPS-stimulated leukocyte was not markedly affected by Gu-4, but the clustering of CD11b was partly inhibited (J *vs*. N). Original magnification, ×630.

We have previously identified CD11b, an integrin on the surface of leukocyte, as a target molecule of Gu-4 [17], but the precise role of Gu-4 in the interfering with the function of CD11b remains largely unknown. Since affinity and avidity regulations are the main mechanisms that regulate the function of CD11b in the process of inflammation [24], [25], we thus examined the expression and distribution of CD11b and its active “I-domain” in the leukocytes isolated from the whole blood by flow cytometry and confocal microscopy. Under normal conditions, CD11b and its I-domain are expressed at a low level and distributed unevenly on the surface of neutrophils (Figure 4A, B; Figure 5B, D). Gu-4 alone did not change the expression and distribution styles of CD11b (Figure 4A, B; Figure 5F, H). However, when cells were exposed to LPS, the amount of CD11b and its I-domain on neutrophils was greatly increased, and both of them redistributed on the cell membrane to form clusters (Figure 4A, B; Figure 5J, L). To the LPS-stimulated cells, Gu-4 showed more potent inhibitory effect on the exposure of CD11b I-domain than on the expression of CD11b itself (Figure 4A, B). Interestingly, changes of redistribution of CD11b (Figure 5N *vs.* J) and CD11b I-domain (Figure 5P *vs.* L) were significantly blocked by Gu-4. We speculated that Gu-4 attenuates leukocyte adhesion and subsequent transendothelial migration through blocking the activation and the formation of clusters of CD11b on the membrane.

### Gu-4 blocks the activation of Syk in LPS-stimulated THP-1 cells

**Figure 6:**
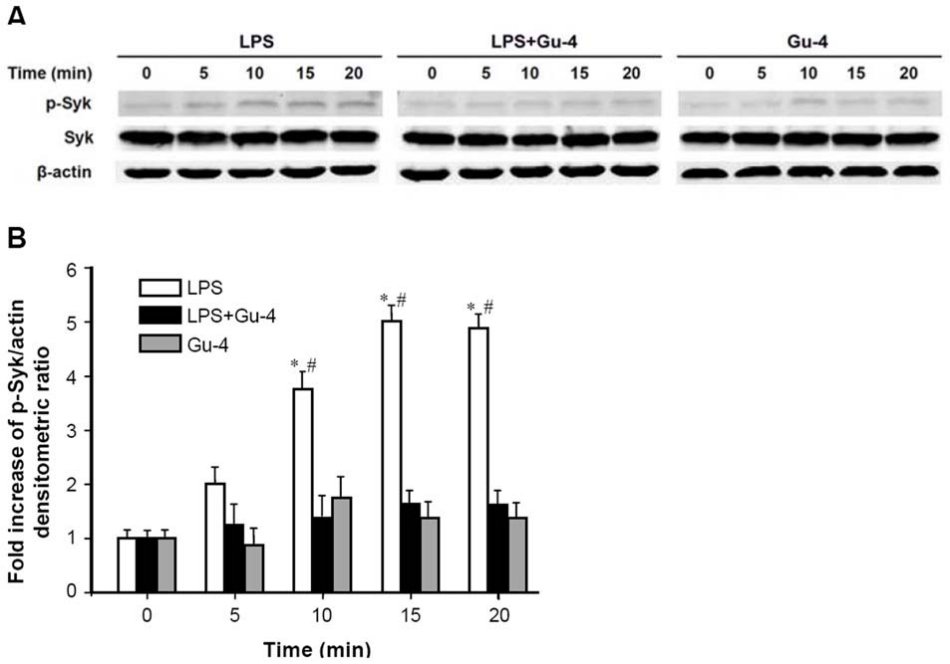
Effects of Gu-4 on activation of Syk in LPS stimulated THP-1 cells. A: Western Blot analyses of Syk and phosphorylated Syk (p-Syk) in THP-1 cells stimulated with LPS alone (100 ng/ml), LPS (100 ng/ml) plus Gu-4 (40 nmol/ml), or Gu-4 alone (40 nmol/ml) for 0 to 20 mins; β-actin serves as a loading control. B: The densitometric analyses showed that LPS-induced increase of phosphorylation of Syk was markedly blocked by Gu-4. These statistical data were from 5 independent experiments. *: p<0.05 *vs.* the LPS + Gu-4 or Gu-4 treated group at indicated time point. #: p<0.05 *vs.* time point of 0 min.

To confirm the potent inhibitory effect of Gu-4 to the activation of CD11b in the LPS-stimulated leukocytes, we further examined the expression of downstream effector responsible for CD11b signaling transduction in the THP-1 cells. By Western blot analysis, we determined the expression of spleen tyrosine kinase (Syk), an important signaling molecule for signal-transduction of integrin outside-in signaling [26]. As shown in Figure 6A and B, the expression level of phosphorylated Syk in the THP-1 cells initiated to increase at time point of 5 min immediately after LPS stimulation and sustained at the later time points (10, 15, 20 min), while Syk protein level was unchanged. However, the LPS-triggered increase of Syk phosphorylation was attenuated by the combined LPS and Gu-4 treatment. Gu-4 treatment alone did not induce Syk activation or Syk upregulation in the THP-1 cells. This result demonstrated that Gu-4 treatment provided strong blockade on the activation of CD11b.

### Gu-4 improves survival in polymicrobial sepsis

**Figure 7:**
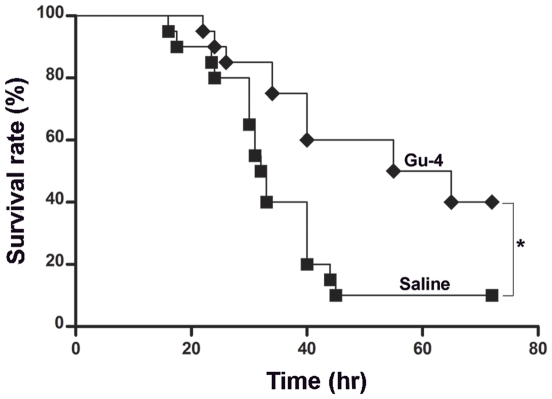
Effect of Gu-4 on survival of septic mice induced by cecal ligation and puncture (CLP). CLP was performed as described in Materials and Methods. Saline or Gu-4 (0.929 mg/kg per 3 hr) treatment were performed separately in two groups of mice subjected to CLP. Data represent the percentages of survival rates at the indicated time points after CLP surgery; n = 20 animals per group. A significant increase of survival was found in the group treated with Gu-4 after CLP surgery compared with the group treated with saline after CLP surgery, *: p<0.05.

The model of cecal ligation and puncture (CLP) is a well established polymicrobial sepsis model, which has been demonstrated to be the most representative animal model of human sepsis [27], [28], we therefore investigated whether Gu-4 also have protective effects on animals after CLP. CLP treatment resulted in a 90% mortality by the end of observation (72 hrs). While using the ED50 dose of Gu-4 for septic mice, administration of Gu-4 at the dose of 0.929 mg/kg per 3 hrs significantly improved the survival of mice after CLP (p<0.05, Gu-4 *vs.* saline) (Figure 7), demonstrating that Gu-4 has an effective therapeutic activity in polymicrobial sepsis, a inflammatory response occurring in humans.

## Discussion

Progressive organ damage and dysfunction occurred in septic patients are closely correlated with systemic leukocyte activation and disseminated leukocyte adhesion [29], [30]. The currently available supportive therapeutic interventions can not prevent or reverse organ damage, which underlined an urgent need for specific therapeutic treatments. In this study, we examined the role of Gu-4, a lactosyl derivative, which had been successfully synthesized and demonstrated to be a specific inhibitor of leukocyte integrin CD11b, in protecting mice underwent endotoxemia or polymicrobial sepsis from mortality and the underlying mechanism involved in this role. We found that this compound greatly inhibited the activation and cluster formation of CD11b on the membrane of LPS-stimulated leukocyte. This result made it understandable that Gu-4 treatment protected animals from mortality and acute lung injury in LPS-induced and CLP-induced septic shock models.

Normally, the inflammation response of innate immune system toward invading pathogens is tightly regulated [31]. However, in the situation like experimental endotoxemia in which the inflammatory stimuli (such as LPS) are intense, inflammatory mediators are produced in an unregulated manner. The cascade of cytokine and chemokine production will lead to uncontrolled leukocyte infiltration and activation that damage the tissue leading to organ dysfunction. Although these events can affect all tissues of the organism, the lung appears to be primary target. In this study, firstly we analyzed the effect of different Gu-4 doses on survival rate. The lowest dose of the compound tested was ineffective in terms of survival benefit, whereas at the incrementally higher doses tested, the statistically significant therapeutic benefit appeared, suggesting that proper and sufficient amount of Gu-4 is necessary to produce the therapeutic effects. Interestingly, at ED50 dose of 0.929 mg/kg as estimated by Probit analysis, Gu-4 did not alter the expression profiles of plasma proinflammatory cytokine TNF-α or anti-inflammatory cytokine IL-10, suggesting that the biological effects of Gu-4 on septic shock did not directly correlated with the inhibition of the early inflammatory mediator release. We thus speculated that Gu-4 might play its roles by being involved in the process of cell adhesion and migration. Subsequently, we analyzed the effects of Gu-4 on leukocytes infiltration histologically in the lung tissues *in vivo* and on adhesion and migration of THP-1 cells *in vitro*. Histological examinations of lung tissues revealed that leukocyte infiltration and injury were markedly attenuated by Gu-4 treatment compared to vehicle treatment. In addition, *in vitro* Gu-4 treatment inhibited the adhesion and migration of LPS-stimulated THP-1 cells to HUVECs. Therefore, it is conceivable that attenuated leukocyte infiltration in lung tissue should be attributed to the attenuated adhesion and migration of leukocytes affected by Gu-4 administration through circulation, thereby attenuating the inflammatory response induced by LPS and protecting animals from septic shock.

Under the condition of shock, the imbalance of oxygen demands and oxygen supply will lead to tissue hypoxia and the subsequent increase of lactate production. The amount of produced lactate is generally believed to be correlate with the total oxygen debt, the magnitude of hypoperfusion, and the severity of shock [32], [33]. One of the proposed mechanisms for the deficient oxygen delivery in sepsis is the occlusion of capillaries [34]. Accumulating evidences to date support that leukocyte is a likely candidate to occlude capillaries [35]. Therefore, we examined the LA level in the lung tissues and found that a significant increase of LA was observed in lung tissues from LPS-stimulated mice but not from LPS-stimulated mice with Gu-4 treatment. The lung administrated with Gu-4 after LPS challenge exhibited the normal architectures and the attenuated leukocyte infiltration. Therefore, it is possible that Gu-4 treatment alleviated the occlusion of capillaries caused by the enhanced adhesion of leukocytes in sepsis. Obviously, these data strengthened our conclusion of the therapeutic values of Gu-4 in treatment of septic shock. In addition, our results also support the notion that early lactate clearance is associated with improved outcome in severe sepsis and septic shock [32], [33].

The firm adhesion of leukocytes to endothelial cells and the transendothelial migration were mainly mediated by β2 integrins and their ligands [11]. These molecules are heterodimers that share a common β subunit (CD18) with distinct α subunits such as CD11b, whose activity is tightly controlled. There are two distinct mechanisms had been proposed to explain the activation of integrin. The first mechanism suggests that alterations in the affinity of integrins lead to enhanced ligand binding. It is believed that a distal N-terminal extracellular “I-domain” is critical for ligand binding in each of these two subunits [24]. The usually embedded “I-domain” of α subunit undergoes a conformation change in response to inflammation, leading to the activation of integrin. The conformation change or exposure of “I-domain” will enhance the affinity of the integrin to its ligand. The second mechanism suggests that the avidity of integrins is increased as a result of increased cluster formation of cell surface receptors [25], [36]. In the present study, we examined the effects of Gu-4 on the affinity and avidity modulations of CD11b. The LPS-induced exposure of CD11b “I-domain” and the cluster formation of CD11b/CD18 were significantly inhibited by Gu-4 treatment. Thus, these results implied a possible underlying mechanism for the inhibitory effect of Gu-4 on leukocyte adhesion, that is, Gu-4 targets to CD11b and inhibits the conformation change of CD11b and its cluster formation, resulting in a weakened adhesion of leukocytes.

It has been reported that the therapeutic uses of ligand mimetic integrin antagonists have paradoxically enhanced integrin function and worsened some clinical outcomes [37]. Because ligand binding merely shifts the conformational equilibrium toward the fully active state of integrin, and the active as well as high-affinity conformation is sufficient to induce outside-in signaling. In addition, through ITAM-coupled activation of the nonreceptor tyrosine kinase Syk pathway, outside-in signaling can regulate a variety of cellular properties such as cell shape, migration, cytotoxicity, growth, and survival. These evidences suggest that antagonists which inducing integrin conformation changes or initiating outside-in integrin signaling are not suitable for clinical use, no matter how effective their antagonistic activities are. While in our study, we showed that the LPS-triggered activation of Syk was potently blocked by Gu-4, demonstrating that Gu-4 binded to CD11b and blocked its activation without touching the outside-in signaling in the cytoplasm. In this sense, we believe that Gu-4 provides more therapeutic value in treatment of sepsis than conventional antagonists against CD11b.

Sepsis and septic shock are very harmful disorders with complicated mechanisms. Endeavour to alleviate the adhesion between leukocyte and endothelial cells has been considered as an alternative way for the treatment of septic shock besides conventionally adopted interventions. Although the understanding of underlying mechanisms by which Gu-4 protects mice from endotoxemia and sepsis requires systemic investigation, our *in vitro* data support the hypothesis that Gu-4 inhibits the leukocyte-endothelial cell adhesion by modulation of the affinity and avidity of leukocyte integrin CD11b and alleviates the inflammatory response and organ injury, thereby providing therapeutic effects to animals. Importantly, our findings shed light on the potential use of Gu-4, an interacting compound to CD11b, in the treatment of sepsis and septic shock.

## Materials and methods

### Ethics statement

This study was carried out in strict accordance with the requirements of Provisions and General Recommendation of Chinese Experimental Animals Administration Legislation and were approved by Science and Technology Department of Jiangsu Province [Permit Number: SYXK (Su) 2010-0003]. All surgeries were performed under sodium pentobarbital anesthesia, and all efforts were made to minimize suffering. Blood samples of healthy donors were purchased from Jiangsu Province Blood Center and approved by the Blood Donation Office of Jiangsu Province and College of Life Science, Nanjing Normal University (Permit Number: 037).

### Preparation of Gu-4

Endotoxin-free lactosyl derivative Gu-4 was prepared in our laboratory and confirmed by nuclear magnetic resonance (NMR), mass-spectrometry (MS), elemental analysis and Limulus Amebocyte Lysate assay.

### Cell culture

Human umbilical vein endothelial cells (HUVECs) obtained from ScienCell were grown in endothelial cell medium (ECM, 1001, ScienCell) with 1% endothelial cell growth supplement (ECGS, 1052, ScienCell). All experiments with HUVECs were performed below 8 passages. THP-1 cells (human acute monocytic leukemia cell line) were purchased from the CBCAS (Cell Bank of the Chinese Academic of Sciences, Shanghai, China) and maintained in RPMI1640 (GIBCO) supplemented with 15% (v/v) fetal bovine serum. All cells were maintained at 37°C in a humidified 5% CO_2_ incubator.

### Effect of Gu-4 in the LPS model of endotoxemia

Eight-week-old ICR mice (Shanghi Laboratory Animal Center, Chinese Academy Sciences, Shanghai, China) were used in this study. Mice were injected *i.p.* with freshly prepared LPS (7.5 mg/ml, Sigma, *Escherichia coli* O111:B4) dissolved in saline solution at the dose of 37.5 mg/kg to induce endotoxemia [38]. All drugs used for treatment were dissolved in saline solution and given through tail vein injection at 30 mins after LPS challenge (0.005 ml/g), saline solution was used as a control treatment. After separately treated with incremental doses of Gu-4 (0.74, 0.88, 1.06, 1.27, or 1.53 mg/kg), β-lactose (0.27 mg/kg) (Sigma, L3750), dexamethasone (1.3 mg/kg) (Jinling Pharmaceutical, China), or saline, the animals were returned to their cages and fed *ad libitum*. The death of animal was determined by rigorous physical examinations, including no response to pain, no corneal reflex and no spontaneous respirations. The survival rate was studied in n = 10 mice in each group. An additional group of mice (n = 10) received Gu-4 alone (10 mg/kg) without LPS treatment was designed as control. A total of 80 mice was used in survival study. In another set of experiments, the ED50 dose of Gu-4 for survival was tested on a number of additional parameters. For the measurements of plasma cytokines or lactic acid (LA) and histological examination of lungs, animals were treated with saline or Gu-4 (0.929 mg/kg) after the LPS challenge, and were killed at 0 hr (baseline), 1 hr, 2 hr, 4 hr, 6 hr, 8 hr and 10 hr (n = 10 mice at each time point), respectively.

### Histological examination

Lung tissues on the right side were isolated from mice and fixed in 10% formalin for 24 hrs at 4°C, embedded in paraffin and then serially sectioned. The sections were stained with hematoxylin and eosin (H&E) and observed under light microscope. Each section was evaluated by two investigators blinded to the treatment of animals. Lung injury was assessed in sections using a four-category scale system, including edema, hemorrhage, leukocyte infiltration and alveolar septal thickening [39], to grade the degree of lung injury in 8 fields. Each category was scored from 0 to 4 (0 = normal; 1≤25%; 2 = 25–50%; 3 = 50–75%; 4≥75%); the sum of each individual scores was used to represent the total lung injury.

### Measurement of LA in lung tissue

Lung tissues on the left side were collected from mice, weighed and immediately homogenized individually with 0.9% saline at a ratio of 1∶9 (w/v) at 4°C. The homogenates were centrifuged (3000 rpm for 10 mins) at 4°C and the supernatants were used for assays. The protein content in the supernatant was determined by Bradford assay [40]. The LA level in the supernatant was measured by using a lactate assay kit (Nanjing Jiancheng Bioengineering Institute, Nanjing, China). The LA level in lung extracts was expressed as millimole LA per gram of protein.

### Measurement of serum levels of TNF-α and IL-10

The concentrations of TNF-α and IL-10 in serum samples were determined simultaneously by using a commercial multiplexing assay kit (CBA kit 552364, BD Biosciences, San Jose, CA). Samples and standards were prepared following the manufacturer's instructions and analyzed on a BD FACScan equipped with BD CellQuest and CBA software (BD Biosciences, Franklin Lakes, NJ, USA).

### Adhesion assay

To examine the effect of Gu-4 on leukocyte-endothelial interaction under static condition, HUVECs (1×10^5^ cells per well) were cultured in a 96-well culture plate (Greiner) to confluence, followed by activation with TNF-α 10 u/ml (Bioworld Technology, Inc. , St. Louis Park, MN, USA) for 24 h. Aliquots of THP-1 cells (1×10^5^ cells per aliquot) were firstly stained with Calcein-AM (8 µM) (C1359, Sigma) for 40 mins at 37°C, followed by 15 mins treatment of LPS (1 µg/ml) or culture media without LPS. After washed for three times, the THP-1 cells were subjected separately to the treatments of Gu-4 (40 nmol/ml), β-lactose (40 nmol/ml), CD11b antibody (4 µg/ml) (301214, Biolegend), or saline for 10 mins. Then, the treated THP-1 cells were co-incubated with HUVECs in HEPES CaMg buffer (0.05 M HEPES, 0.15 M NaCl, 1 mM CaCl_2_, 1 mM MgCl_2_, pH 7.4) for 20 mins at 37°C. The non-adherent cells were removed by washing with 1×isotonic phosphate buffered saline (PBS) (pH 6.0). The adherent cells were quantified by fluorescence measurement with a fluorescence plate reader (Bio-Tek SynergyII) at 485 nm/528 nm. The mean fluorescence intensity (MFI) of triplicate wells was used for statistical analysis.

### Transwell migration assay

To examine the effects of Gu-4 treatment on the migration behavior of leukocyte, *in vitro* chemotaxis assays were performed using 3.0 µm pore-size Transwell inserts (Millipore, PISP12R48) in 24-well plates (Corning). HUVECs were cultured in inserts until confluent, followed by stimulation with 10 u/ml of TNF-α for 24 hrs. After aspiration of the culture medium from the insert without disturbing the endothelial monolayer, Calcein-AM stained and LPS treated THP-1 cells were subjected to the treatments as described in adhesion assay, and then put in the inserts (3×10^5^ cells/300 µl per insert). The lower well contained 500 µl of serum-free RPMI 1640 with 100 ng/ml IL-8 (208-IL, R&D Systems, Minneapolis, MN). The plates were incubated at 37°C for 4 hrs before the inserts were carefully removed. The migrated cells were determined by reading the fluorescence intensity of the lower chamber with a fluorescence plate reader (Bio-Tek SynergyII) at 485 nm/528 nm. The MFI of triplicate wells was used for statistical analysis. The absence of adherent cells to the undersurface of the inserts was ascertained by light microscopy.

### Measurement of CD11b expression by flow cytometry

To examine the effects of Gu-4 on the expression of CD11b, heparinized whole blood samples were collected intravenously from healthy donors. Briefly, blood samples were aliquoted (200 µl per aliquot) and co-incubated with Gu-4 (40 nmol/ml) and LPS (100 ng/ml) for 30 mins. Then the aliquots were further co-incubated separately with PE-labeled anti-CD11b polyclonal antibody (sc-1186, Santa Cruz, CA, USA) or PE-conjugated monoclonal antibody CBRM1/5 (12-0113, eBioscience, San Diego, CA, USA) that binds to the I domain of CD11b for 10 mins. Red blood cells in samples were lysed with lysis buffer (KHCO_3_ 1 g, NH_4_Cl 8.3 g, EDTA-Na_2_ 37 mg, add sterile water to 1,000 ml, adjust pH to 7.2 with HCl) at a ratio of 1∶10 (v/v). After centrifugation at 1500 rpm for 5 mins, leukocytes were pelleted and resuspended in 300 µl PBS. At least 5,000 cells were analyzed using the Guava ExpressPro application (Guava CytoSoft Software, version 4.2) on a Guava EasyCyte Flow Cytometer (Guava Technologies). Non-specific fluorescence background was evaluated by comparing the fluorescence detected from the cells incubated with antibodies having crossmatched isotypes.

### Observation of CD11b distribution

CD11b distribution on leukocytes was observed by a Carl Zeiss LSM 700 laser scanning confocal microscope (LSCM, Car Zeiss Microimaging, Inc., Thornwood, NY, USA). Leukocytes obtained and treated as described in flow cytometry assay were further incubated with Qdot 655 secondary antibody (Q11022MP) (Invitrogen, Eugene, OR, USA) for 1 hr at room temperature in the dark. The cells were collected by centrifugation at 1000× rpm for 5 mins and washed for three times with PBS, then resuspended in 300 µl PBS. A single drop of cell suspension was placed onto a slide and a cover slip was applied on the top. Images were obtained by using 488 nm Diode laser combined with a Plan-Apochromat 63×/1.40 Oil DIC M27 oil immersion objective lens. Only those of cells with diameters about 10 µm were imaged. The images captured by LSCM were analyzed by ZEN 2008 Light Edition software. The two-dimensional intensity distribution of an image was displayed in a pseudo 3D mode (2.5D filled view).

### Western blotting

THP-1 cells that cultured in 24-well plate (3×10^5^ per well) were stimulated with LPS (100 ng/ml), Gu-4 (40 nmol/ml), or both for 5, 10, 15, 20 min, respectively. Cells were then rinsed twice with ice-cold PBS, and lysed with a lysis buffer (20 mM Tris, 135 mM NaCl, 2 mM EDTA, 2 mM DTT, 25 mM β-glycerophosphate, 2 mM sodium pyrophosphate, 10% glycerol, 1% Triton X-100, 1 mM sodium orthovanadate, 10 mM NaF, pH 7.5) containing 10 µg/ml aprotinin, 10 µg/ml leupeptin, and 1 mM phenylmethylsulfonyl fluoride (PMSF). Lysates were cleared by centrifugation (15 mins at 15,000×g, 4°C), diluted with 2×SDS sample buffer (0.5 M Tris-HCl (pH 6.8) 2 ml, 10% SDS 4 ml, glycerol 2 ml, β-mercaptoethanol 1 ml, bromophenol blue 1.5 mg, add deionized water to 10 ml, store under 4°C), and boiled. Protein extracts were resolved on 12% SDS polyacrylamide gel and immunoblotting was performed as described previously [41]. Proteins of interest were visualized by probing the blots with primary antibody against Syk (MAB88906) (Chemicon international Inc., Temecula, CA, USA) or against phospho-Syk (Tyr525/526) (#2711) (Cell Signaling Technology, Inc., Danvers, MA, USA) and the secondary antibody flurophore-conjugated IRDye800 (LI-COR Biosciences, Lincoln, NE), respectively. Antibodies were diluted to a final concentration of 0.5 to 1 µg/ml. The signal intensity was quantified by LI-COR Odyssey Analysis software. Densitometric analysis of the expression level of phosphorylated Syk on the immunoblots was normalized by that of β-actin. Representative immunoblots from five independent experiments are shown.

### Effect of Gu-4 in the CLP model of polymicrobial sepsis

Mice were anesthetized with pentobarbital sodium (30 mg/kg) (Sinopharm Chemical Reagent Co., Ltd., Shanghai, China). Under aseptic conditions, a 1.5-cm midline laparotomy was performed to allow exposure of the cecum. The cecum was tightly ligated with a 3-0 silk suture placed at its base, and was perforated twice with an 18-gauge needle. Then the cecum was gently squeezed to extrude a small amount of feces from the perforation sites. The laparotomy was closed with 4-0 silk sutures after the cecum was returned to the peritoneal cavity [42]. Ten mice underwent laparotomy but not cecum ligation and puncture were taken as controls. The administration of saline solution or Gu-4 (0.929 mg/kg) to animals through i.p. (n = 20 mice in each group) was started after 3 hrs of surgery, and was continuously given every 3 hrs for 72 hrs or until the death of individual. The survival rates were monitored within 72 hrs.

### Statistical analysis

Data are presented as mean ± SD. Statistical analysis was performed by statistics package for social science (SPSS) of 13.0-version. One way analysis of variance followed by Student-Newman-Keuls multiple comparison tests. Survival of mice was analyzed with Kaplan–Meier survival analysis with the log-rank test for between-group comparisons. p<0.05 was considered statistically significant. ED50 of Gu-4 for LPS endotoxemia model was determined by Probit analysis.

## References

[1] Winters BD, Eberlein M, Leung J, Needham DM, Pronovost PJ (2010). Long-term mortality and quality of life in sepsis: a systematic review.. Crit Care Med.

[2] McCuskey RS, Urbaschek R, Urbaschek B (1996). The microcirculation during endotoxemia.. Cardiovasc Res.

[3] De Petrocellis L, Di Marzo V (2010). Non-CB1, non-CB2 receptors for endocannabinoids, plant cannabinoids, and synthetic cannabimimetics: focus on G-protein-coupled receptors and transient receptor potential channels.. J Neuroimmune Pharmacol.

[4] Hollenberg SM, Guglielmi M, Parrillo JE (2007). Discordance between microvascular permeability and leukocyte dynamics in septic inducible nitric oxide synthase deficient mice.. Crit Care.

[5] Lehmann C, Georgiew A, Weber M, Birnbaum J, Kox WJ (2001). Reduction in intestinal leukocyte adherence in rat experimental endotoxemia by treatment with the 21-aminosteroid U-74389G.. Intensive Care Med.

[6] Chishti AD, Shenton BK, Kirby JA, Baudouin SV (2004). Neutrophil chemotaxis and receptor expression in clinical septic shock.. Intensive care medicine.

[7] Schnare M, Barton GM, Holt AC, Takeda K, Akira S (2001). Toll-like receptors control activation of adaptive immune responses.. Nat Immunol.

[8] Hodgson JC (2006). Endotoxin and mammalian host responses during experimental disease.. J Comp Pathol.

[9] Luster AD, Alon R, von Andrian UH (2005). Immune cell migration in inflammation: present and future therapeutic targets.. Nat Immunol.

[10] Chandra A, Enkhbaatar P, Nakano Y, Traber LD, Traber DL (2006). Sepsis: emerging role of nitric oxide and selectins.. Clinics (Sao Paulo).

[11] Hu G, Vogel SM, Schwartz DE, Malik AB, Minshall RD (2008). Intercellular adhesion molecule-1-dependent neutrophil adhesion to endothelial cells induces caveolae-mediated pulmonary vascular hyperpermeability.. Circ Res.

[12] Alencar NM, Oliveira RS, Figueiredo JG, Cavalcante IJ, Matos MP (2010). An anti-inflammatory lectin from Luetzelburgia auriculata seeds inhibits adhesion and rolling of leukocytes and modulates histamine and PGE2 action in acute inflammation models.. Inflamm Res.

[13] Decking J, Mayer A, Petrow P, Seiffge D, Karbowski A (2001). Antibodies to PECAM-1 and glucocorticoids reduce leukocyte adhesion in adjuvant arthritis of the rat knee synovium in vivo.. Inflamm Res.

[14] Joshi A, Bauer R, Kuebler P, White M, Leddy C (2006). An overview of the pharmacokinetics and pharmacodynamics of efalizumab: a monoclonal antibody approved for use in psoriasis.. J Clin Pharmacol.

[15] Engel C, Brunkhorst FM, Bone HG, Brunkhorst R, Gerlach H (2007). Epidemiology of sepsis in Germany: results from a national prospective multicenter study.. Intensive care medicine.

[16] Levy MM, Dellinger RP, Townsend SR, Linde-Zwirble WT, Marshall JC (2010). The Surviving Sepsis Campaign: results of an international guideline-based performance improvement program targeting severe sepsis.. Intensive Care Med.

[17] Zhao Z, Li Q, Hu J, Li Z, Liu J (2009). Lactosyl derivatives function in a rat model of severe burn shock by acting as antagonists against CD11b of integrin on leukocytes.. Glycoconjugate journal.

[18] Ghezzi P, Cerami A (2004). Tumor necrosis factor as a pharmacological target.. Methods in molecular medicine.

[19] Pruitt JH, Welborn MB, Edwards PD, Harward TR, Seeger JW (1996). Increased soluble interleukin-1 type II receptor concentrations in postoperative patients and in patients with sepsis syndrome.. Blood.

[20] Rittirsch D, Flierl MA, Ward PA (2008). Harmful molecular mechanisms in sepsis.. Nature Reviews Immunology.

[21] Karima R, Matsumoto S, Higashi H, Matsushima K (1999). The molecular pathogenesis of endotoxic shock and organ failure.. Molecular medicine today.

[22] Gutierrez G, Wulf M (1996). Lactic acidosis in sepsis: a commentary.. Intensive care medicine.

[23] Haji-Michael PG, Ladrière L, Sener A, Vincent JL, Malaisse WJ (1999). Leukocyte glycolysis and lactate output in animal sepsis and ex vivo human blood.. Metabolism.

[24] Lee JO, Rieu P, Arnaout MA, Liddington R (1995). Crystal structure of the A domain from the a subunit of integrin CR3 (CD11 b/CD18).. Cell.

[25] van Kooyk Y, Figdor CG (2000). Avidity regulation of integrins: the driving force in leukocyte adhesion.. Current opinion in cell biology.

[26] Hirahashi J, Mekala D, Van Ziffle J, Xiao L, Saffaripour S (2006). Mac-1 signaling via Src-family and Syk kinases results in elastase-dependent thrombohemorrhagic vasculopathy.. Immunity.

[27] Baker C, Chaudry I, Gaines H, Baue A (1983). Evaluation of factors affecting mortality rate after sepsis in a murine cecal ligation and puncture model.. Surgery.

[28] Hubbard WJ, Choudhry M, Schwacha MG, Kerby JD, Rue LW (2005). Cecal ligation and puncture.. Shock.

[29] Liu L, Kubes P (2003). Molecular mechanisms of leukocyte recruitment: organ-specific mechanisms of action.. Thrombosis and haemostasis.

[30] Ploppa A, Schmidt V, Hientz A, Reutershan J, Haeberle HA (2010). Mechanisms of leukocyte distribution during sepsis: an experimental study on the interdependence of cell activation, shear stress and endothelial injury.. Critical Care.

[31] Mogensen TH (2009). Pathogen recognition and inflammatory signaling in innate immune defenses.. Clinical microbiology reviews.

[32] Nguyen HB, Rivers EP, Knoblich BP, Jacobsen G, Muzzin A (2004). Early lactate clearance is associated with improved outcome in severe sepsis and septic shock*.. Critical care medicine.

[33] Nguyen HB, Loomba M, Yang JJ, Jacobsen G, Shah K (2010). Early lactate clearance is associated with biomarkers of inflammation, coagulation, apoptosis, organ dysfunction and mortality in severe sepsis and septic shock.. Journal of Inflammation.

[34] Ince C (2005). The microcirculation is the motor of sepsis.. CRITICAL CARE-LONDON-.

[35] Ellis CG, Jagger J, Sharpe M (2005). The microcirculation as a functional system.. CRITICAL CARE-LONDON-.

[36] Kim M, Carman CV, Yang W, Salas A, Springer TA (2004). The primacy of affinity over clustering in regulation of adhesiveness of the integrin |ÁL|Â2.. The Journal of cell biology.

[37] Lefort CT, Hyun YM, Schultz JB, Law FY, Waugh RE (2009). Outside-in signal transmission by conformational changes in integrin Mac-1.. The Journal of Immunology.

[38] Liu Z, Fan Y, Wang Y, Han C, Pan Y (2008). Dipyrithione inhibits lipopolysaccharide-induced iNOS and COX-2 up-regulation in macrophages and protects against endotoxic shock in mice.. FEBS letters.

[39] Murakami K, Bjertnaes LJ, Schmalstieg FC, McGuire R, Cox RA (2002). A novel animal model of sepsis after acute lung injury in sheep.. Crit Care Med.

[40] Bradford MM (1976). A rapid and sensitive method for the quantitation of microgram quantities of protein utilizing the principle of protein-dye binding.. Analytical biochemistry.

[41] Adler V, Yin Z, Tew KD, Ronai Z (1999). Role of redox potential and reactive oxygen species in stress signaling.. Oncogene.

[42] Soriano FG, Lorigados CB, Pacher P, Szabo C (2011). Effects of a potent peroxynitrite decomposition catalyst in murine models of endotoxemia and sepsis.. Shock.

